# Meta-analysis of correlates of HPV vaccine acceptability among men: supporting vaccine implementation science

**DOI:** 10.1186/1742-4690-9-S2-P229

**Published:** 2012-09-13

**Authors:** PA Newman, C Logie, K Asakura

**Affiliations:** 1 University of Toronto, Toronto, Canada

## Background

Understanding ubiquitous research-to-practice gaps in uptake of adult vaccines, particularly those for sexually transmitted infections (e.g., suboptimal human papillomavirus [HPV] vaccine uptake among men in the US), may provide evidence to support the successful dissemination of future HIV vaccines. To this end, we assessed rates of HPV vaccine acceptability and factors correlated with HPV vaccine acceptability among men.

## Methods

We used a comprehensive search strategy across multiple electronic databases to locate studies that examined rates and/or correlates of HPV vaccine acceptability. The search strategy had no date or language restrictions. Search keywords included vaccine, acceptability and all terms for human papillomavirus. We calculated mean HPV vaccine acceptability scores across studies. We conducted meta-analysis using a random effects model on cross-sectional studies reporting correlates of HPV vaccine acceptability. All studies were assessed for risk of bias.

## Results

Of 301 identified studies, 22 met inclusion criteria. Across 18 studies (n=7787), weighted mean HPV vaccine acceptability = 52.3 (SD 17.5) (100-point scale). HPV vaccine acceptability was significantly higher among gay/bisexual/MSM (65.1, SD 15.1) versus heterosexual men (47.3, SD 14.7). Among 11 studies (n=4064) included in meta-analyses, perceived HPV vaccine benefits and doctor recommendation had medium effect sizes, and the following factors had small effect sizes on HPV vaccine acceptability: anticipatory regret, perceived effectiveness, fear of side effects, perceived partner support for vaccination, perceived susceptibility to HPV, number of lifetime sexual partners, having a current sex partner, Hepatitis B vaccine uptake, smoking cigarettes, HPV awareness, knowledge and non-white ethnicity.

## Conclusion

Public health campaigns tailored for men that promote positive HPV vaccine attitudes, HPV knowledge and risk awareness, and healthcare provider education, may support HPV vaccine acceptability for men; these factors may be instructive in planning for future HIV vaccine dissemination. Future investigations employing rigorous designs, including intervention studies, are needed to support effective vaccine promotion among men.

